# Rhomboid protease Rhbdl2 regulates macrophage recruitment and wound regeneration in zebrafish

**DOI:** 10.64898/2026.02.13.705804

**Published:** 2026-02-15

**Authors:** Saroj Gourkanti, Gayathri Ramakrishnan, Yazmin Munoz, Rosa M. Chavez, Jacqueline Cheung, Jan Dohnálek, Taylor J. Schoen, Katie Martin, Matthew Lovett-Barron, Thomas Whisenant, Kvido Strisovsky, Sonya E. Neal

**Affiliations:** 1 School of Biological Sciences, Department of Cell and Developmental Biology, University of California San Diego, La Jolla, CA 92093, USA; 2 School of Biological Sciences, Department of Neurobiology, University of California San Diego, La Jolla, CA 92093, USA; 3 Institute of Organic Chemistry and Biochemistry, Czech Academy of Sciences, Flemingovo n. 2, Prague, 1660 100, Czech Republic; 4 University of Chemistry and Technology, Prague, Technická 5, Prague, Czech Republic; 5 Center for Computational Biology and Bioinformatics, University of California San Diego, La Jolla, Ca 92093, USA; 6 Howard Hughes Medical Institute, Chevy Chase, MD 20815, USA; 7 Lead contact

**Keywords:** rhomboid protease, wounding healing, Rhbdl2, regeneration, neutrophil, macrophage

## Abstract

Tissue regeneration requires tight control of immune cell behavior, yet the mechanisms that restrain immune-driven regenerative responses remain poorly defined. Here, we identify the rhomboid intramembrane serine protease Rhbdl2 as a critical regulator of regeneration in zebrafish. We generated *rhbdl2* mutants by CRISPR-Cas9 and found that it does not affect normal development, but triggers enhanced regenerative growth following injury, accompanied by increased macrophage accumulation at the wound site, which is accompanied by increased early apoptosis and proliferation. Proteomic analyses reveal increased Rac2 protein levels in *rhbdl2* mutants, indicating dysregulated immune signaling. Functionally, Rac2 morpholino oligonucleotides-mediated knockdown in *rhbdl2* mutant larvae suppresses the elevated macrophage recruitment and enhanced tissue regenerative phenotype. Together, these findings uncover Rhbdl2 as an immune checkpoint that constrains macrophage-driven enhanced regeneration, with vast implications for inflammatory disease, fibrosis, and tumor–immune interactions.

## Introduction

Millions of people worldwide will suffer from chronic, non-healing wounds over the course of their lifetime, representing a major and growing clinical burden(Sen et al., 2009). Effective wound repair requires the precise integration of spatially and temporally coordinated signaling events that operate at both the single-cell and multicellular level, encompassing epithelial cells, immune populations, and the extracellular matrix (Gao et al., 2024; Rousselle et al., 2019; Sorg et al., 2017). These processes are orchestrated by tightly regulated ligand–receptor signaling networks that control inflammation, cell migration, proliferation, and tissue remodeling (Liu & Fang, 2025; Mendonce et al., 2025). While regenerative wound healing shares components with homeostatic developmental pathways, the regenerative processes of wound healing also possess unique regulators that functions in different stages(Khan et al., 2018).

The innate immune system plays an important role in modulating the wound healing process by orchestrating the recruitment and behavior of leukocytes, including macrophages and neutrophils(De Oliveira et al., 2016; Phillips et al., 2020). These cells work by balancing pro- and anti-inflammatory signaling at the wound. Following injury, stress cues initiated by neighboring keratinocytes rapidly recruit neutrophils, which amplify and propagate inflammatory signaling throughout the wound site (Wilgus et al., 2013; Xu et al., 2024; Zhu et al., 2021). These early events subsequently promote the recruitment of macrophages, which function to combat infection and drive pro-regenerative signaling program (Krzyszczyk et al., 2018; Minutti et al., 2017). Dysregulated leukocyte recruitment or aberrant immune cell behavior can impair wound resolution, contributing to chronic wounds and increased susceptibility to infection (Miskolci et al., 2019a; Schilrreff & Alexiev, 2022). While cell-intrinsic regulators such as Rac family GTPases are essential for leukocyte migration and function (Deng et al., 2011; Mishra et al., 2023; Rosowski et al., 2016a), emerging studies continues to reveal novel regulators that modulate extrinsic recruitment signaling during tissue repair (Golenberg et al., 2020; Houseright et al., 2020; Karnam et al., 2023; Soto et al., 2025; Tsourouktsoglou et al., 2020).

The intramembrane serine protease Rhbdl2 has recently emerged as an attractive candidate for mediating wound healing (Cheng et al., 2011a; Johnson et al., 2017). It is a member of the highly conserved rhomboid superfamily (Adrain et al., 2011a; Freeman, 2014; Strisovsky, 2013). The rhomboid superfamily comprises intramembrane proteases, which function in many biological processes such as membrane protein homeostasis(Began et al., 2020; Bhaduri et al., 2025; Fleig et al., 2012; Grieve et al., 2021), intercellular signaling (Lemieux et al., 2007; Stevenson et al., 2007; Urban et al., 2002), parasitic invasion (Baker et al., 2006; Brossier et al., 2005; Gandhi et al., 2020), and protein trafficking(Knopf et al., 2024; Paschkowsky et al., 2016; Wunderle et al., 2016). Rhomboid intramembrane proteases cleave substrates within the lipid bilayer through a conserved serine-histidine catalytic dyad (Ha et al., 2013; Tichá et al., 2018). While their overall structural architecture of rhomboid proteases is highly conserved, individual family members exhibit remarkable substrate specificity, targeting and cleaving distinct and nonoverlapping repertoires of substrates (Bhaduri et al., 2022; Gourkanti et al., 2025). Notably, Rhbdl2 localizes to the cell surface and is enriched along the plasma membrane(Lohi et al., 2004), positioning it ideally to regulate ligand availability and receptor activation in response to tissue injury (Adrain et al., 2011b; Noy et al., 2016; Pascall & Brown, 2004).

Current knowledge of Rhbdl2 function is largely based upon the identification of its substrate repertoire via proteomic approaches, which have shown that Rhbdl2 cleaves critical factors of wound healing such as thrombomodulin, DDR1 and Spint1 (Johnson et al., 2017). Moreover, *in vitro* wound assays in Rhbdl2 mutant HaCaT cells shows a significant delay in wound repair (Cheng et al., 2011). Importantly, previous characterization of Rhbdl2 function thus far relied heavily on *in vitro* cell culture studies, and no vertebrate knockout model has been established based on perinatal lethality observed in mice knockouts (mousephenotype.org).

Zebrafish have the remarkable ability to regenerate various tissues such as heart, spinal cord, and skin and these processes are conserved in humans(Arjmand et al., 2020; Cigliola et al., 2020; Greenspan et al., 2024). Furthermore, there are well-established wound repair assays in zebrafish along with genetics tools and established transgenic lines to allow for an extensive exploration of Rhbdl2 function on cellular behavior in a regenerative context(Miskolci et al., 2019a). Therefore, zebrafish is a well-suited vertebrate model for robust systematic analysis of Rhbdl2. To that end, we established that zebrafish possess a single, highly conserved ortholog of Rhbdl2. Using an *in vitro* cleavage assay in HaCaT cells, we demonstrate that zebrafish Rhbdl2 retains enzymatic activity comparable to its human counterpart, confirming functional conservation across species. Accordingly, we generated a *rhbdl2* mutant zebrafish line and used wound assays, fluorescent imaging, and proteomic profiling to characterize its role in vertebrate wound healing. While loss of Rhbdl2 does not perturb embryonic development or adult viability, *rhbdl2* mutants exhibit a striking phenotype during tissue repair, characterized by enhanced regeneration following injury. To uncover the molecular basis of this response, we performed unbiased global proteomic profiling and identified a significant post-transcriptional increase in Rac2 protein levels in *rhbdl2* mutants, a key regulator of leukocyte dynamics. Live imaging and quantitative analyses in *rhbdl2* mutant larvae revealed robust accumulation of macrophages and, to a lesser extent, neutrophils at the wound site, actively driving the enhanced regenerative response observed in *rhbdl2* mutants. Together, these findings identify Rhbdl2 as a previously unrecognized regulator of injury-induced tissue regeneration, acting through modulation of immune cell dynamics.

## Results

### Zebrafish rhbdl2 functions as a canonical rhomboid protease

To investigate the role of the rhomboid protease Rhbdl2 in zebrafish, we characterized the annotated zebrafish gene *rhbdl2*. Our analysis identified transcripts with 8 exons (205, 202, 204), 6 exons (203), and 7 exons (201) (Fig. S1A). The *rhbdl2* gene encodes a 294-amino-acid protein (GenBank: AAI64054.1) that is highly conserved among vertebrates, with homologous sequences present in lower eukaryotic and prokaryotic organisms. Sequence alignment of human and zebrafish Rhbdl2 polypeptides from the canonical transcript 205 demonstrated a high level of identity (64%), where zebrafish Rhbdl2 possesses the conserved serine-histidine dyad essential for proteolytic activity. Additionally, key rhomboid motifs, including WR and GxxxG, which are conserved across all rhomboid proteins (Kandel & Neal, 2020; Lemberg & Freeman, 2007) , are retained in zebrafish Rhbdl2 (Fig. S1B). Structural modeling using AlphaFold demonstrated that zebrafish Rhbdl2 retains the same fold as the human Rhbdl2 homolog (Fig. S1C). Additionally, *in situ* hybridization data from the ZFIN database, together with single-cell RNA sequencing data from Zebrahub, indicate that *rhbdl2* is expressed in the zebrafish epidermis throughout development beginning at 2 days post fertilization (dpf) (data not shown). To assess the proteolytic activity of zebrafish Rhbdl2, we subcloned its open reading frame (ORF) into a pcDNA3.1 expression vector and transfected HEK293 cells with either human or zebrafish Rhbdl2. Expression of both human and zebrafish Rhbdl2 was readily detected in cell lysates. Using a cleavage-based assay, when cells are transfected with either human or zebrafish Rhbdl2, we observed the release of a cleavage product of the well-characterized human Rhbdl2 substrate Spint1 into the culture medium, whereas cells transfected with empty vector showed only full-length Spint1 retained in the lysate (Fig. S1D). Based on this finding, we concluded that zebrafish *rhbdl2* functions as a canonical rhomboid protease, maintaining conserved activity similar to its mammalian counterpart.

### An RNA-less rhbdl2−/− allele does not impact zebrafish development

**Fig. 1.**
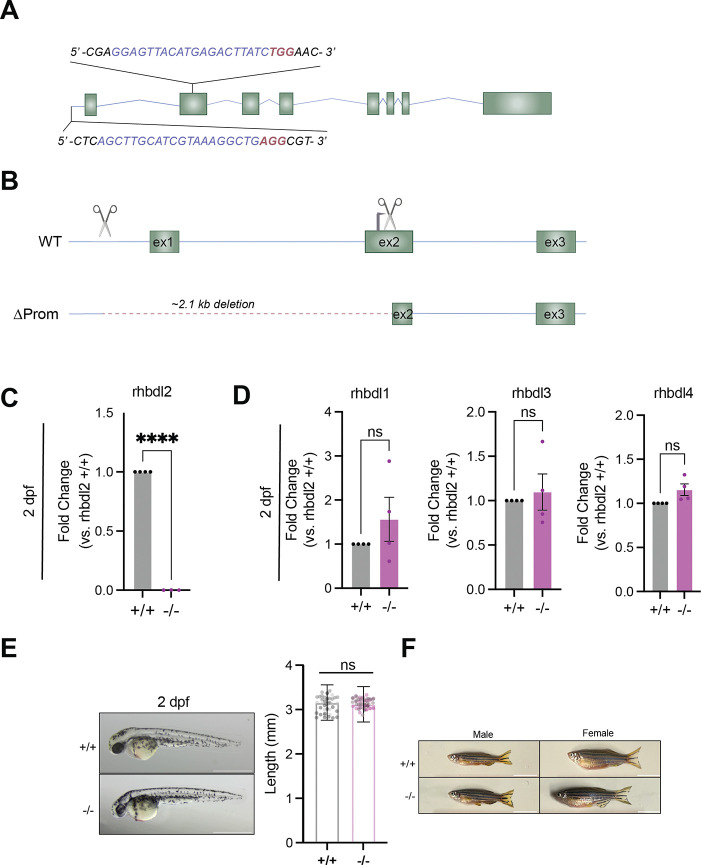
*rhbdl2* promoterless knockout zebrafish model does not display developmental defects. **(A)** Schematic of *rhbdl2* gene with 5’ gRNA sequence and exon 2 gRNA sequence highlighted for CRISPR/Cas9 mutagenesis. Sequence of gRNA is displayed in blue and protospacer adjacent motif displayed in red. **(B)** Sequence comparison of WT and ΔProm knockout allele illustrating 2.1 kb deletion. CRISPR/Cas9 guide sites highlighted by scissors and translation start site shown by black arrow. **(C)** RT-qPCR of *rhbdl2 exon*4/5 and **(D)** rhomboid family proteases *rhbdl1, rhbdl3,* and *rhbdl4* on pooled homozygous cousin larvae from WT and ΔProm homozygous sibling incrosses. Data is from four pooled replicates with n=10–20 larvae per sample. Means and SEM reported and one-sample t-test performed. ****, p<0.0001. **(E)** Representative full-body brightfield images of 2dpf larvae, WT cousin (top) and *rhbdl2*^−/−^ (bottom). Scale bar = 1 mm. Quantification of full body length between WT and *rhbdl2*^−/−^ larvae at 2dpf. Data is from three independent replicates with lsmeans (± SEM) reported and p-values calculated by ANOVA with Tukey’s multiple comparisons, n = 43 (+/+), 46 (−/−). **(F)** Representative full-body images of 6mpf adult zebrafish cousins with males (left) and females (right) indicated. Data from two independent replicates and scale bar = 100 mm.

To investigate the function of the *rhbdl2* gene, we generated an RNA-null allele using CRISPR-Cas9 to minimize compensatory mechanisms. Two guide RNAs (gRNAs) were designed to delete 2.1 kb of the upstream promoter region, exon 1, and the translation start site of exon 2, effectively preventing mRNA production (Fig. 1–B). The resulting *rhbdl2* heterozygous founders were identified by Sanger sequencing and outcrossed to generate stable heterozygote animals. These fish were subsequently inbred to obtain homozygous mutants (*rhbdl2*−/−). qPCR analysis confirmed the absence of *rhbdl2* mRNA expression in zebrafish larvae at 2 dpf (Fig. 1C). Because the rhomboid superfamily comprises multiple rhomboid proteases, we next sought to determine whether *rhbdl2*−/− zebrafish triggers redundancy or genetic compensation by other rhomboid family members. We quantified the mRNA levels of *rhbdl1*, *rhbdl3*, and *rhbdl4* (Fig. 1D). Notably, despite the genetic loss of *rhbdl2*, the expression levels of other rhomboid family members remained unchanged. Next, we examined whether *rhbdl2*−/− zebrafish exhibited any morphological abnormalities. Across different developmental stages, no visible defects were observed in 2 dpf larvae as well as in both adult females and males (Fig. 1E–F). Collectively, these findings indicate that the loss of *rhbdl2* does not impact zebrafish development.

### Loss of Rhbdl2 induces enhanced regeneration following injury

**Fig. 2.**
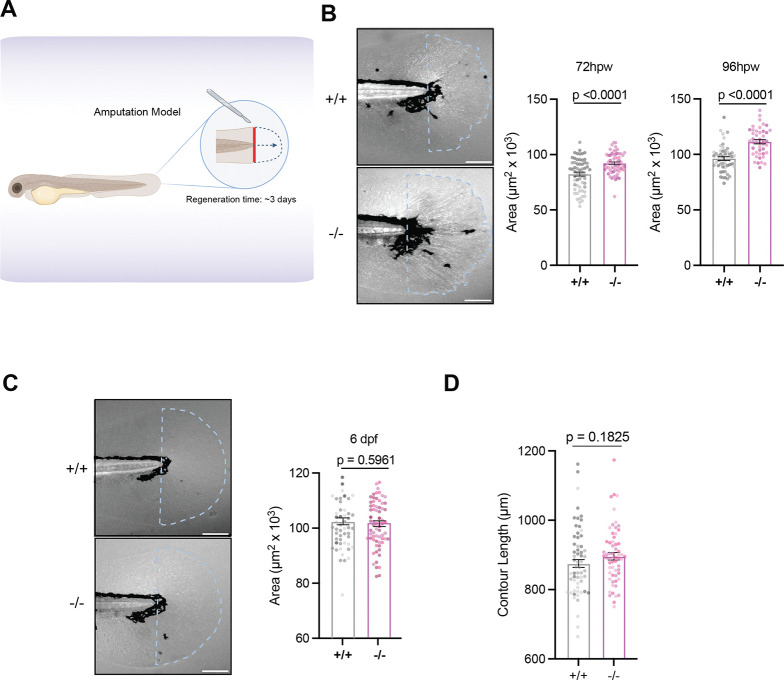
Loss of Rhbdl2 leads to enhancedregeneration. **(A)** Cartoon displaying transection wound assay conducted on 3dpf larvae. Transections performed at tip of notochord and general regeneration time is considered complete at 3 days post wounding (dpw). Fin areas were measured from tip of notochord. **(B)** Representative brightfield images of regeneration on 72hpw tails. Quantification of regenerated area at 72hpw and 96hpw from three independent replicates. 72hpw: n = 59 (+/+), 61 (−/−), 96hpw: n = 52 (+/+), 46 (−/−). **(C)** Representative brightfield images of unwounded tails age-matched at 6dpf. Quantification of developmental fin area of 6dpf larvae from three independent replicates, n = 52 (+/+), 68 (−/−). **(D)** Quantification of contour length in 72hpw wounds in 2B. Contour length calculated as wound perimeter minus tail width at notochord tip. Data is from three independent replicates with lsmeans (± SEM) reported, and p-values calculated by ANOVA with Tukey’s multiple comparisons. Scale bars = 100 μm.

To examine the role of Rhbdl2 in regeneration, we performed tail transections on 3 dpf larvae at the tip of the notochord. Homozygous *rhbdl2* mutant and WT offspring were generated from incrosses of F3 or F4 adult WT or *rhbdl2*−/− siblings. Both r*hbdl2*−/− and WT cousins underwent tail transection, and the regenerated area (measured from the vessel edge to the caudal edge of the tail fin) was evaluated beginning at 3 days post-wounding (dpw) (Fig. 2A). Notably, *rhbdl2*−/− mutants displayed a significant increase in regenerated area at 3 dpw compared to WT cousins at 72 hpw and 96 hpw (Fig. 2B). Given Rhbdl2’s role in caudal fin regeneration, we next examined whether it is required for fin development. Whole tail measurements of 6 dpf larvae that were age-matched to wounding experiments revealed no significant differences between WT and *rhbdl2*−/− mutant (Fig. 2C). These findings suggest that Rhbdl2 is required for the regulation of caudal fin regeneration but not for its development. To determine whether loss of Rhbdl2 alters large-scale tissue architecture during regeneration, we quantified contour length as a proxy for collagen projections, a metric that reflects extracellular matrix organization and has been shown to correlate with effective regenerative capacity(LeBert et al., 2018; Miskolci et al., 2019a). This analysis revealed no significant difference in contour length between WT and *rhbdl2−/−* mutant (Fig. 2D). Given that fin contour length was indistinguishable between WT and *rhbdl2*−/− larvae, we next asked whether loss of Rhbdl2 affects more subtle aspects of ECM organization during regeneration. We utilized a cell-impermeable ECM-specific dye, Rhobo6, to visualize the collagen matrix in the regenerating fin at 72 hpw (Fiore et al., 2025). Quantitative phenotypic scoring of collagen recovery did not reveal statistically significant differences among genotypes (Fig. S2–B). Together, these data indicate that loss of Rhbdl2 does not result in aberrant regeneration but instead is associated with enhanced wound repair.

### Rhbdl2-deficient larvae have increased wound-induced proliferation and apoptosis

**Fig. 3.**
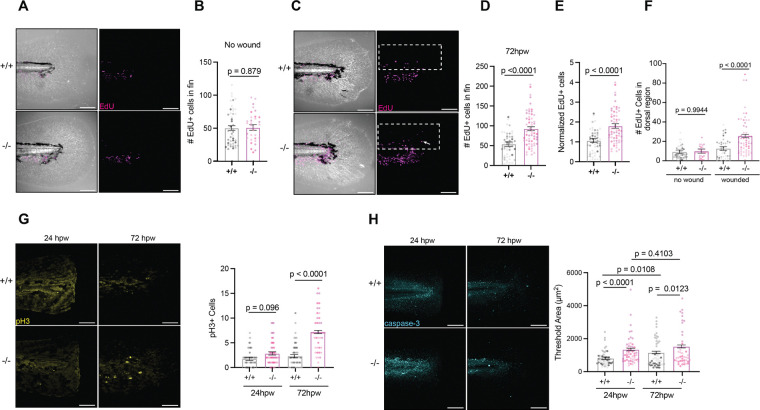
Loss of Rhbdl2 leads to increased proliferation and early apoptosis during regeneration. **(A and C)** Representative images of developmental unwounded (A) or 72hpw (C) fins pulsed with EdU for 6h. Merged images of brightfield images and EdU (magenta) shown on left with single EdU (magenta) image shown on right. Dorsal region highlighted by white box in (C). **(B and D)** Quantification of EdU-positive cells in unwounded (B) and 72hpw (D). **(E)** Number of EdU-positive cells in wounded fin normalized to respective unwounded conditions. **(F)** Quantification of EdU-positive cells within dorsal area of the fin. All EdU data are from four pooled independent replicates and cell counts were calculated from the region at tip of caudal blood loop through tail fin (no wound, n = 46 (+/+), 32 (−/−); 72hpw, n = 47 (+/+), 58 (−/−)). **(G)** Representative images of phospho-H3 (yellow) stained tails at 24hpw (left) and 72hpw (right). Quantification of pH3-positive nuclei shown on right. **(H)** Representative images of active-Caspase3 (cyan) stained tails at 24hpw (left) and 72hpw (right). Quantification of active-Caspase3 threshold area shown on right. Data is from three independent experiments (24hpw, n = 54 (+/+), 54 (−/−); 72hpw, n = 53 (+/+), n= 42 (−/−)). Lsmeans (± SEM) reported and p-values calculated by ANOVA with Tukey’s multiple comparisons for all experiments. Scale bars = 100 μm.

Regeneration is a dynamic and coordinated process where the balance between cell proliferation and apoptosis is crucial for restoring the correct tissue architecture following injury (Aragona et al., 2017; Justynski et al., 2023; Zanca et al., 2022). To assess proliferative activity during fin regeneration, we performed 6-hour EdU pulse labeling on 6 dpf larvae that were either left unwounded or subjected to tail transection and collected at 72 hpw. Representative merged brightfield and EdU (*magenta*) images are shown, with single EdU channels displayed in the right panel for clarity (Fig. 3A,C). In both developmental (unwounded) and regenerating fins, EdU-positive cells were detected predominantly within the distal fin fold region. Quantification of EdU-positive nuclei revealed no significant difference in proliferative index between WT and *rhbdl2*−/− larvae under unwounded conditions (Fig. 3B). However, following injury, there was a robust increase in EdU incorporation throughout the regenerating fin (Fig. 3D–E), indicating elevated proliferative activity during tissue repair. To further quantify wound-induced proliferation, we focused on the dorsal region of the tail, as developmental proliferation is largely localized to the ventral fin (Golenberg et al., 2020). Within the dorsal region (*white box in Fig. 3C*), *rhbdl2−/−* mutants exhibited a significant increase in proliferation following wounding compared with their WT cousins, whereas no differences were observed between unwounded WT and mutant fins (Fig. 3F). These results suggest that Rhbdl2 specifically influences the regenerative proliferative response following injury. To complement our EdU incorporation analyses, we next assessed mitotic activity by co-staining wounded larvae with phospho-histone H3 (pH3, yellow), a marker for cells primed to undergo mitosis (Fig. 3G). Consistent with our EdU findings, quantitative imaging revealed a significant increase in pH3-positive cells at 72 hpw in wound regions, but not at earlier time points (Fig. 3G). To determine whether impaired regeneration in *rhbdl2−/−* larvae was also associated with altered cell death dynamics, we next examined apoptosis by staining for active Caspase-3 at 24 and 72 hours hpw (Fig. 3H). Representative images show active Caspase-3–positive signal (cyan) concentrated at higher levels during early regenerative stages. Quantification of the active Caspase-3–positive area revealed a significant increase in apoptosis in *rhbdl2−/−* cousins compared with WT at both 24 and 72 hpw (Fig. 3H). Importantly, this temporal pattern indicates that the increase in cell proliferation occurs subsequent to the onset of elevated apoptosis. Together, our findings indicate that loss of Rhbdl2 elicits an excessive regenerative response characterized by increased cell proliferation and apoptosis.

### Proteome profile of rhbdl2−/− zebrafish reveals alterations in Rac protein

**Fig. 4.**
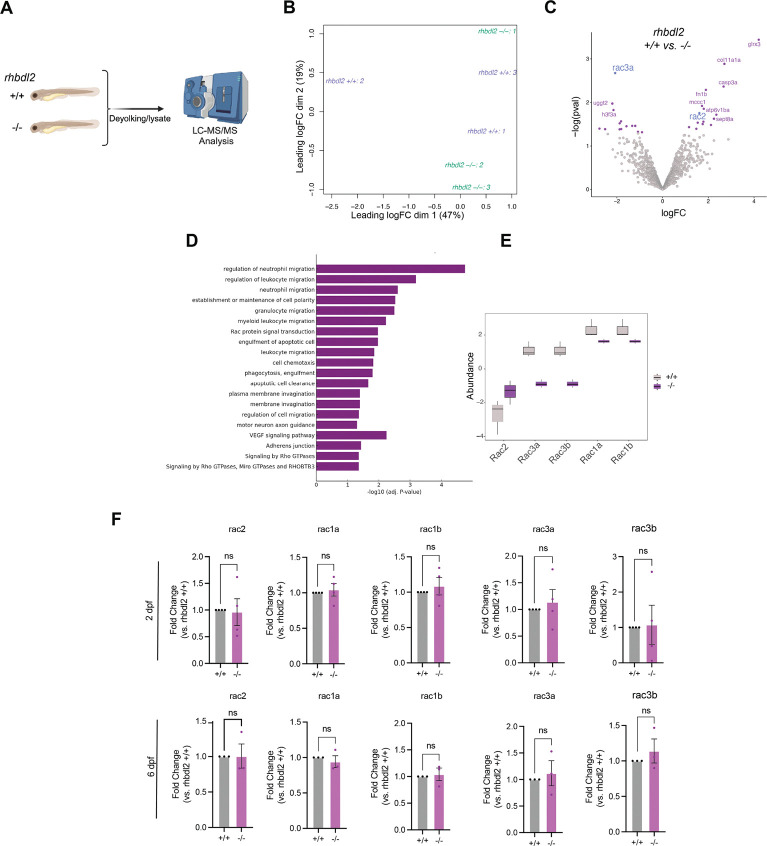
*rhbdl2*^*−/−*^ mutants display differences in Rac protein levels. **(A)** Schematic depicting larval preparation of protein lysates for LC-MS/MS analysis. Data is from three independent experiments with n = ~50 larvae per sample **(B)** Multi-dimensional scaling (MDS) plot of the replicate WT (purple) and *rhbdl2*^*−/−*^ (green) samples. The x-axis shows the separation of the samples on the first dimension and accounts for 47% of the variability in the data, while the y-axis shows the second dimension and accounts for 19% of the variability. **(C)** Volcano plot of the abundance of proteins in the limma comparison of WT and *rhbdl2*^*−/−*^ samples. The x-axis shows the log fold change, and the y-axis represents the negative log of the comparison p-value. Proteins with a significant p-value (less than 0.05) are colored purple, and all others are colored white. The top 20 proteins (by p-value) are labeled. (**D)** Pathway enrichment analysis using gProfiler against cellular component GO terms. **(E)** Boxplot of the Rac proteins show the abundance of each protein in the WT (white) and *rhbdl2*^*−/−*^ (purple) samples. The box represents the interquartile range (IQR, 25%−75%) with the median represented by a black line. The whiskers represent the range +/− 1.5x IQR. **(F)** RT-qPCR of Rac family genes on pooled homozygous cousin larvae from WT and ΔProm homozygous sibling incrosses. Data is from four pooled replicates with n=10–20 larvae per sample. Means and SEM reported and unpaired t-test performed.

Because Rhbdl2 functions as an intramembrane protease that directly cleaves and regulates the abundance of its substrates, we sought to determine how loss of Rhbdl2 impacts the global proteome *in vivo*. To obtain an unbiased, systems-level view of protein-level changes associated with Rhbdl2 deficiency, we performed label-free quantitative proteomics on zebrafish larvae. We focused our analysis at 2 dpf, a developmental stage at which Rhbdl2 expression is well established to be in epithelial tissues, based on *in situ* hybridization data from ZFIN. We utilized label-free quantitative proteomics to analyze global proteome changes in 2 dpf zebrafish larvae, comparing WT (n=150) and *rhbdl2−/−* mutant (n=150) groups (Fig. 4A). Multi-dimensional scaling analysis revealed that *rhbdl2−/−* mutant replicates clustered more closely together than WT (Fig. 4B). However, a heatmap (Fig. S3A), clustered based on protein abundance, suggests a high degree of similarity in *rhbdl2−/−* mutant samples or WT larvae samples. Mass spectrometry identified a total of 8,932 swiss-prot protein id proteins, with 2,425 meeting significance thresholds for inclusion in statistical analysis. Among them, 48 proteins were differentially expressed, with 26 lower protein abundance and 22 exhibiting higher protein abundance in *rhbdl2−/−* mutants compared to WT (Fig. 4C, Fig. S4A). Notably, among the differentially expressed genes, *bcam, cdh2, fras1, sccphdb, glg1a, glg1b, and plxnb2a* encode factors involved in epithelial homeostasis and were also significantly enriched in a previous *in vitro* screen (Fig. S3B) (Johnson et al., 2017). Gene ontology (GO) analysis indicated that the differentially regulated proteins were primarily associated with biological processes such as neutrophil and leukocyte migration, Rac protein signal transduction, apoptotic cell engulfment, and cell chemotaxis (Fig. 4D).

Rho GTPases are crucial regulators of innate immune cell functions, including migration and phagocytosis. The Rac subfamily of Rho GTPases consists of Rac1, Rac2, Rac3, and RhoG, each differing in expression patterns. Rac1 is ubiquitously expressed, Rac2 is primarily found in hematopoietic cell lineages, and Rac3 is predominantly expressed in neural tissue. Rac2 constitutes 90% of the Rac protein in immune cells, where Rac1 and Rac2 share both redundant and distinct functions(Deng et al., 2011). Moreover, Rac proteins are well-characterized for playing a vital role in immune cell motility (Rosowski et al., 2016b). Proteomics analysis using box plots confirmed that Rac1a, Rac1b, Rac3a, and Rac3b exhibited lower protein abundance, whereas Rac2 has higher protein abundance in *rhbdl2−/−* mutants compared to WT (Fig. 4E). RT-qPCR analysis of *Rac* gene mRNA levels showed no significant changes, suggesting that alterations in Rac levels occur at the protein level rather than through transcriptional regulation (Fig. 4F). Collectively, our proteomic analysis suggests that Rhbdl2 affects Rac GTPases at a post-transcriptional level, underscoring a potential role for Rhbld2 in innate immunity.

### Loss of Rhbdl2 increases late-phase macrophage accumulation at wounds during tailfin regeneration

Our proteomic analyses suggest that Rhbdl2 modulates leukocyte migration, an important process contributing to overall wound recovery. Neutrophils are among the first immune cells to respond to epithelial injury, triggering inflammatory signals that promote the recruitment and proliferation of macrophages and other cell types necessary for pro-regenerative wound healing. However, excessive neutrophil and macrophage accumulation or failure to migrate to the wound site can impair proper tissue repair (Miskolci et al., 2019). Accordingly, *Tg(lyz:GFP; mpeg1:dsRed)* reporter fish were crossed into the *rhbdl2−/−* mutant background to enable simultaneous visualization of neutrophil and macrophage recruitment to tail wounds during regeneration.

**Fig. 5.**
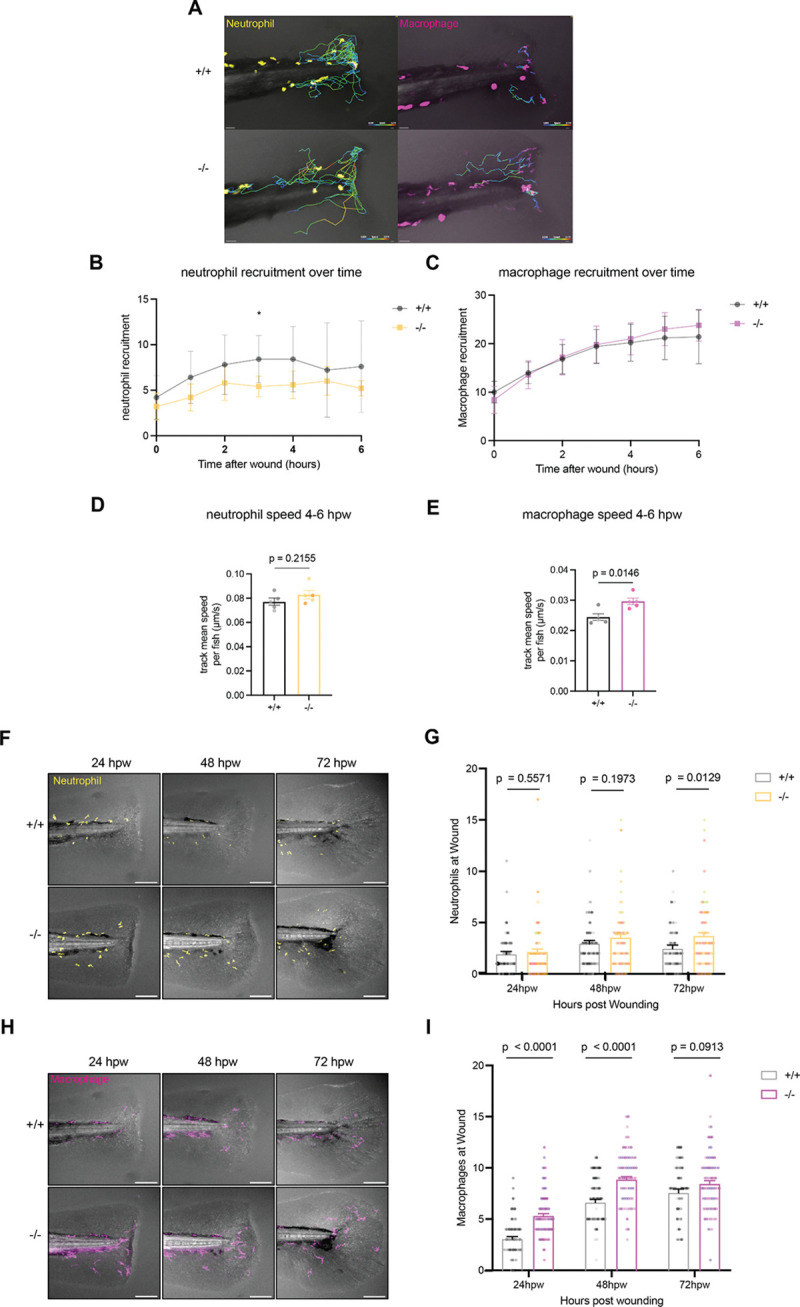
Loss of Rhbdl2 elicits increased retention of macrophages to tailfin wound. **(A)** Representative serial images from 6hpw time-lapse imaging comparing WT and *rhbdl2−/−* in (Tg(*lyz:GFP;mpeg1:dsRed)*) background; see Video 1. Lines representing neutrophils (top) or macrophages (bottom) tracks over time shown with warmer colors indicating faster speed. Data is from two independent replicates with n = 5 (+/+), 5 (−/−). **(B-C)** Quantification of number of neutrophils (top) or macrophages (bottom) in wound region distal to caudal vein loop over course of time lapse imaging. **(D-E)** Quantification of instantaneous speed of neutrophils (D) and macrophages (E) during later half (4–6hpw) of time-lapse imaging; Lsmeans (± SEM) reported and p-values calculated by ANOVA with Tukey’s multiple comparisons for all experiments. Scale bars = 30 μm. **(F-H)** Representative images of leukocytes at wounded tails from 24–72hpw, visualized with GFP-labeled neutrophils (F) and dsRed-labeled macrophages (H) (Tg(*lyz:GFP;mpeg1:dsRed)*) merged with brightfield image. **(E)** Quantification of neutrophils at wound. **(G-I)** Quantification of neutrophils and macrophages at wound. All data from four independent replicates (24hpw, n = 66 (+/+), 70 (−/−) ; 48hpw, n = 66 (+/+), 64 (−/−) ; 72hpw, n = 56 (+/+), 71 (−/−)).

We first focused on the initial recruitment of leukocytes to the wound using time-lapse microscopy from 1–6 hpw in the *Tg(lyz:GFP; mpeg1:dsRed)* background (Fig. 5A). As expected, we observed a steady increase in both neutrophil and macrophage recruitment to the wound between 1 and 6 hpw. In *rhbdl2*−/− mutants, neither neutrophil nor macrophage recruitment was significantly impaired compared to WT. However, we observed a trend toward increased macrophage recruitment at 5–6 hpw, although this difference did not reach statistical significance (Fig. 5A–C). In addition, we assessed neutrophil and macrophage motility toward the wound by measuring their migration speed and detected no significant differences between genotypes (Fig. 5D–E). We next turned our attention to later stages of leukocyte recruitment. Transection wound assays were performed on transgenic siblings, and tails were fixed and imaged at 24, 48, and 72 hpw (Fig. 5F). Neutrophil recruitment was comparable between WT and *rhbdl2*−/− larvae at 24 and 48 hpw, with only a modest elevation at 72 hpw in the mutant, in line with previous reports indicating that neutrophils primarily function during the early inflammatory stage of wounding (Fig. 5G). In contrast to neutrophils, macrophage recruitment to the wound was markedly increased in *rhbdl2*−/− mutants at 24 and 48 hpw, followed by normalization by 72 hpw, indicating a prolonged but transient macrophage accumulation during the later stages of regenerative response (Fig. 5H–I).

To determine whether these phenotypes reflected changes in leukocyte abundance, we quantified total neutrophils and macrophages in whole larvae. Neutrophil numbers were modestly reduced in *rhbdl2*−/− mutants at 2 days post-fertilization (dpf), but were indistinguishable from WT by 3 dpf (Fig. S5A–B). In contrast, total macrophage numbers did not differ between WT and *rhbdl2*−/− mutants at either 2 or 3 dpf (Fig. S5C–D). Together, these data indicate that Rhbdl2 primarily modulates macrophage accumulation during the later phases of wound repair, rather than altering leukocyte development.

### Rac2 mediates Rhbdl2-dependent macrophage recruitment and regeneration

**Fig. 6.**
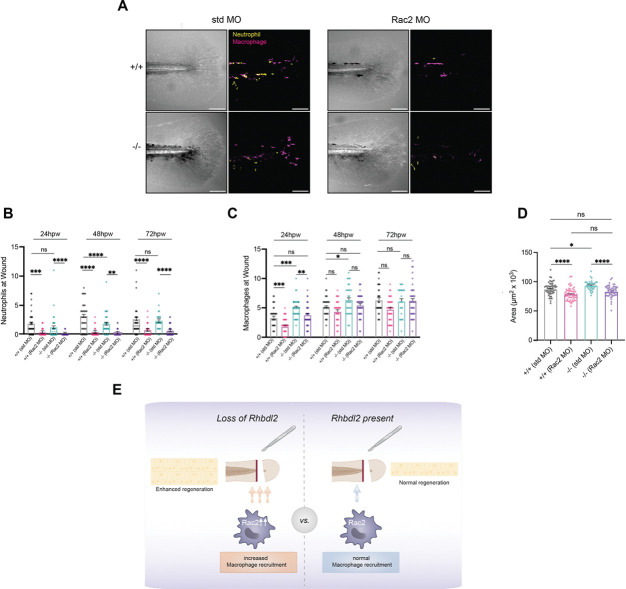
Rac2 ablation in *rhbdl2* mutants rescues regeneration and macrophage recruitment phenotype. **(A)** Representative images of WT or *rhbdl2*^*−/−*^ in (Tg(*lyz:GFP;mpeg1:dsRed)*) background injected with either standard or Rac2 MO. Brightfield images (left) shown alongside merged neutrophil (yellow) and macrophage (magenta, right) images at 72hpw. **(B and C)** Quantification of neutrophils (B) or macrophages (C) at wound sites in Rac2 MO injected samples from 24–72hpw. **(D)** Quantification of regenerated area in Rac2 MO injected samples at 72hpw. Data is from three independent experiments, (+/+: n = 51 (std MO), 56 (Rac2 MO); −/−: n= 40 (std MO), 52 (Rac2 MO)). Lsmeans (± SEM) reported and p-values calculated by ANOVA with Tukey’s multiple comparisons for all experiments. Scale bar = 100 μm. **(E)** Cartoon depicting the in vivo model of Rhbdl2-mediated regeneration. Injury is induced below the notochord in the tail fin. Loss of Rhbdl2 leads to elevated Rac2 protein levels and increased macrophage accumulation at the wound site, resulting in enhanced regenerative outgrowth.

Live cell tracking of macrophages in Rhbdl2-deficient larvae revealed a significant increase in macrophage recruitment to the wound site during later phases of wound healing. Proteomic analyses identified Rac2 as upregulated upon loss of Rhbdl2, suggesting that aberrant Rac2 signaling may underlie these changes in macrophage recruitment. We therefore hypothesized that in *rhbdl2−/−* larvae, morpholino-mediated knockdown of Rac2 would restore macrophage recruitment to a WT-like state during wound regeneration. We employed a translation-blocking Rac2 morpholino previously validated in the literature, which has been shown to reduce both neutrophil and macrophage recruitment to wound site(Deng et al., 2011). Indeed, Rac2 morpholino treatment in WT and *rhbdl2−/−* mutants significantly impaired neutrophil recruitment to the wound at all examined time points (24, 48, and 72 hpw) compared to control morpholino, confirming effective Rac2 knockdown (Fig. 6A–B). Given that Rhbdl2 primarily influences macrophage recruitment during late stage wound repair, we focused our analysis on macrophages. Consistent with earlier reports (Rosowski et al., 2016b), depletion of Rac2 in WT larvae impaired macrophage recruitment to wounds (Fig. 6A, C). As expected, at 24 and 48 hpw, *rhbdl2−/−* mutants injected with control morpholino exhibited a significant increase in macrophage recruitment at the wound compared with WT control. Strikingly, Rac2 knockdown in the *rhbdl2−/−* mutants significantly suppressed this phenotype, restoring macrophage recruitment to levels indistinguishable from WT (Fig.6A, C). At later time points (48 and 72 hpw), macrophage recruitment converged across genotypes and morpholino conditions. Together, these results suggest that Rhbd2-dependent effect of macrophage recruitment during wound repair is mediated through Rac2.

We hypothesized that Rac2-dependent macrophage recruitment drives the enhanced regenerative response observed in *rhbdl2*^*−/−*^ mutants. To test whether elevated Rac2 levels is required for this phenotype, we knocked down Rac2 in the *rhbdl2*^*−/−*^ background. As expected, *rhbdl2*^*−/−*^ + control MO exhibited a significant increase in wound-associated area compared with WT + control MO, while WT +Rac2 MO caused a decrease in regenerative area in comparison to WT+ control (Fig. 6D). Strikingly, *rhbdl2*^*−/−*^ + Rac2 MO significantly suppressed the enhanced regeneration phenotype, restoring wound area measurements to levels indistinguishable from WT (Fig. 6D). Together, these results indicate that Rac2 contributes to enhanced wound regeneration in Rhbdl2-deficient larvae and support a model in which Rhbdl2 constrains regenerative growth through Rac2-dependent regulation of macrophage recruitment.

## Discussion

In this study, we identify the rhomboid intramembrane protease Rhbdl2 as a previously unrecognized regulator of wound-induced inflammation and regeneration in zebrafish. Although Rhbdl2 is dispensable for normal development and fin growth, its loss is associated with enhanced regenerative outgrowth following injury, accompanied by heightened proliferation and apoptosis within the regenerating tissue. Through unbiased proteomic profiling and *in vivo* live cell tracking and imaging, we uncover a central role for Rhbdl2 in shaping macrophage recruitment during wound repair. Specifically, Rhbdl2-deficient larvae exhibit temporally dysregulated immune responses, marked by increased macrophage recruitment at later stages, without corresponding changes in total leukocyte numbers. Mechanistically, our data implicate aberrant Rac2 signaling downstream of Rhbld2 as a key driver of these phenotypes, as Rac2 protein levels are elevated in *rhbdl2*^*−/−*^ mutant*s* and MO-mediated knockdown of Rac2 normalizes macrophage recruitment and suppresses enhanced regeneration (Fig. 6E). Together, these findings position Rhbdl2 as a critical modulator of immune cell behavior that constrains regenerative growth.

Previous *in vitro* studies have implicated Rhbdl2 in wound repair, as loss of Rhbdl2 function in HaCaT keratinocytes impairs scratch wound closure, suggesting a pro-repair role in epithelial cell migration (Cheng et al., 2011a; Johnson et al., 2017). In contrast, our *in vivo* analyses reveal that loss of Rhbdl2 in zebrafish associated with enhanced regeneration following injury. This apparent discrepancy is unlikely to reflect genetic compensation or redundancy, as we generated a promoterless, RNA-null *rhbdl2* allele and confirmed the complete absence of *rhbdl2* mRNA. Moreover, expression levels of other rhomboid family members remained unchanged in Rhbdl2-deficient larvae, ruling out compensatory upregulation as an explanation for the phenotype. Instead, our findings highlight the importance of studying Rhbdl2 function within a multicellular, physiological context. Unlike *in vitro* scratch assays, which primarily capture cell-autonomous migratory behavior, the zebrafish regeneration model allows simultaneous interrogation of epithelial repair, immune cell dynamics, and tissue-scale regenerative responses. Our data indicate that Rhbdl2 plays a critical role in coordinating leukocyte behavior, particularly macrophage recruitment, during wound repair, a layer of regulation that is absent in isolated cell culture systems.

We show that gross accumulation of macrophages at the wound site is associated with enhanced regeneration in Rhbdl2-deficient larvae. At first glance, this finding appears to contrast with prior studies in zebrafish demonstrating that persistent accumulation of pro-inflammatory (M1-like) macrophages impairs regeneration rather than enhancing it(Spencer et al., 2026). However, in *rhbdl2*−/− mutants, we observe enhanced regeneration occurs in the context of heightened macrophage accumulation, suggesting that the regenerative outcome is shaped not simply by macrophage persistent accumulation, but by the functional state of these cells. Supporting this interpretation, a recent study in zebrafish larvae reported that loss of the lipid-sensing receptor Gpr132b promotes a pro-resolving, pro-regenerative macrophage response characterized by increased macrophage accumulation coupled with reduced inflammatory signaling(Soto et al., 2025). Notably, this immune profile phenocopies the enhanced regenerative response observed in Rhbdl2-deficient larvae. Together, these findings suggest that macrophage accumulation can promote regeneration when coupled to a resolving or pro-regenerative activation state, whereas sustained pro-inflammatory macrophage signaling is detrimental to tissue repair. Thus, Rhbdl2 may contribute to restricting macrophage polarization toward an M2-like state during regeneration, helping to balance tissue growth and repair, an area that warrants further investigation.

Rhbdl2 is an intramembrane protease known to cleave membrane-embedded receptors and signaling proteins, thereby releasing soluble signaling fragments into intracellular, luminal, or extracellular compartments. Prior *in vitro* studies have demonstrated that Rhbdl2 cleaves the CRAC channel subunit Orai1to control its abundance, with Rhbdl2 loss resulting in aberrant calcium signaling(Grieve et al., 2021). Given emerging evidence that Orai1-dependent calcium signaling influences macrophage dynamics and functional states during wound healing, an important avenue for future investigation will be to determine how Orai1-mediated calcium signaling integrates with the Rhbdl2–Rac2 axis in regulating macrophage behavior and regenerative outcomes. The additional Rhbdl2 substrate examined in this study, Spint1, is also a key regulator of wound healing. In zebrafish, Spint1 knockdown leads to chronic inflammation and disrupted skin architecture (Mathias et al., 2007). Consistent with these defects, Spint1 mutants exhibit heightened leukocyte recruitment and increased proliferation of basal keratinocytes (Carney et al., 2007), phenotypes that closely mirror those observed in our study. EGFR signaling likewise plays a central role in regulating keratinocyte proliferation and migration during wound regeneration (Pastore et al., 2008; Repertinger et al., 2004), but the potential influence of Rhbdl2 on modulating EGFR activity is unclear. Overexpressed Rhbdl2 was shown to cleave EGF and secrete its bioactive form that can activate EGFR signaling (Adrain et al., 2011b), while overexpressed Rhbdl2 can cleave overexpressed EGFR mediating its degradation(Liao & Carpenter, 2012). These two effects would have opposing directions, and the role of endogenous Rhbdl2 in modulating EGFR warrants further investigation. Despite lack of conclusive information, loss of Rhbdl2 may permit enhanced proliferative signaling during wound repair since Rhbdl2 and its described substrates Spint1 and EGFR have both been implicated in promoting cell proliferation and migration across multiple cancer contexts (Chen et al., 2022; Gómez-Abenza et al., 2019; Normanno et al., 2006). How mechanistically Rhbdl2 differentially regulates regenerative versus pathological growth programs in vivo and the extent to which macrophages contribute to these outcomes remains an important question for future investigation.

Rhbdl2 is expressed predominantly in the epidermis during development, as revealed by in situ hybridization and single-cell RNA-sequencing analyses. Accordingly, our current working model posits that during wound repair, Rhbdl2 functions primarily within epithelial cells to regulate Rac2 levels in macrophages in a cell extrinsic manner. This model is consistent with prior studies demonstrating that macrophages are key drivers of Rac activation in the contexts of both wound healing and tumor invasion (Ramakrishnan et al., 2025). Rac2 activity is tightly regulated by its dynamic cycling between membrane-associated active and cytosolic inactive states, processes controlled by guanine nucleotide exchange factors (GEFs), GTPase-activating proteins (GAPs), and scaffolding proteins(Bos et al., 2007). We speculate that Rhbdl2-dependent intramembrane proteolysis of one or more such regulatory components in epithelial cells may generate downstream signals that alter Rac2 membrane residence time and stability, thereby elevating Rac2 protein levels in the absence of Rhbdl2. Notably, our model does not exclude the possibility that Rhbdl2 also functions directly within macrophages to regulate Rac2 stability or signaling. Dissecting the cell-type–specific contributions of Rhbdl2 and identifying its relevant substrates will be important directions for future work.

Beyond its role in zebrafish regeneration, our findings suggest that Rhbdl2 functions as an immune checkpoint that limits macrophage-driven tissue regeneration. In pathological contexts, dysregulated macrophage recruitment and polarization are central drivers of chronic inflammation, fibrosis, and tumor progression. The ability of Rhbdl2 to restrain Rac2-dependent macrophage accumulation and promote appropriate resolution of the regenerative response positions this protease as a potential molecular brake on maladaptive wound-healing programs. Rhbdl2 therefore functions as a suppressor of regenerative responses by restraining macrophage accumulation and limiting excessive tissue growth. Loss or inhibition of Rhbdl2 activity enhances macrophage persistence at wound sites and promotes regenerative outgrowth, suggesting that transient suppression of Rhbdl2 could be leveraged therapeutically to improve wound healing in contexts where repair is impaired. More broadly, our work identifies intramembrane proteolysis as an unexpected but powerful mechanism by which immune cell behavior is regulated, opening new avenues for therapeutic intervention in inflammatory, fibrotic, and cancer-associated pathologies.

## Materials and Methods

### Zebrafish maintenance and handling

All zebrafish work follows protocols approved by the Institutional Animal Care and Use Committee (IACUC) at the University of California, San Diego (#S20060). Wild-type zebrafish AB lines were used, as well as the previously published transgenic lines Tg(*lyz:EGFP*) (Hall et al. 2007) and Tg(*mpeg1:dsRed*) (Ellett et al. 2011). Adult fish were maintained on a 14-h/ 10-h light/dark schedule. Following breeding, fertilized embryos were maintained in E3 medium. To prevent pigment formation, larvae were maintained in E3 containing 0.2 mM N-phenylthiourea (PTU; Sigma-Aldrich) beginning at 24 h post fertilization. Prior to all experimental procedures, larvae were anesthetized in E3 water containing 0.2 mg/mL Tricaine (ethyl 3-aminobenzoate; Sigma-Aldrich).

### Zebrafish and human RHBDL2 alignment

Predicted transcripts and protein sequences of RHBDL2 are listed in Table 1. Transcript annotations are from GRCz11 for zebrafish with their corresponding IDs from GRCz10 listed in Fig. S1 when applicable. A MUSCLE alignment was generated in Jalview using the protein sequences of mature human RHBDL2 (UniProtKB accession no. Q9NX52; residues 1–303) and mature zebrafish RHBDL2 (UniprotKB accession no. F1QAX9; residues 1–293; Uniprot Consortium 2017). The 191 identical residues of human and zebrafish RHBDL2 (65% sequence identity) were mapped onto the predicted AlphaFold model of human RHBDL2 (Jumper et al 2021).

### Tissue culture and Western blotting

HEK293T cells were incubated in Dulbecco’s modified Eagle’s medium (DMEM) supplemented with 10% FBS at 37°C and 5%(v/v) CO_2_ humidified incubator. All genes were cloned into pcDNA3.1 vector as described (Johnson et al., 2017)and sequence-verified. One day before transfection, HEK293T cells were seeded into a 6-well plate at the density of 5 × 10^5^ cells per well. The transfection reaction was prepared in 100 μl serum-free DMEM (SFM) with 3 μl of FuGENE 6, 600 ng of Spint1 expression plasmid and 300 ng of either human or danio RHBDL2 expression plasmids, respectively. One day after transfection, cells were washed by PBS, and incubated in 750 μl of SFM for another 24 hours. The conditioned SFM was harvested, centrifuged at 2000 × g to remove residual detached cells and debris, and proteins were precipitated by trichloroacetic acid as described(Johnson et al., 2017). Cell lysates were harvested by lysing the attached cell monolayers in 150 μl SDS PAGE sample buffer per well. Protein samples were then analyzed by SDS PAGE and Western blotting, and imaged as described(Johnson et al., 2017).

### Generation of *rhbdl2* mutant line and genotyping

Zebrafish mutants were generated using CRISPR/Cas9 single-cell injections. gRNAs for zebrafish Rhbdl2 (ENSDART00000183149.1) were designed using CRISPRscan (Moreno-Mateos et al. 2015) to target 5’ upstream of exon 1 and within exon 2. The 5’ target sequence was 5’ – AGCTTGCATCGTAAAGGCTG - 3’ while the exon 2 target sequence was 5’ – GGAGTTACATGAGACTTATC – 3’. The gRNAs and Cas9 protein (Integrated DNA Technologies) were prepared by manufacturer’s instructions and combined to a final RNP concentration of 1.5 μM. The RNP solution was injected into one-cell AB embryos in a 1 nL volume. To confirm genome editing, injected 2dpf embryos had genomic DNA extracted and amplified using the primers listed below before being sent out for Sanger sequencing: Rhbdl2 F: 5’ – GTGACACTGGAGCAGCCTAATG- 3’, and Rhbdl2 R: 5’ – CTTTTCAGCTTTCTTTCCAGCCTTC- 3’.

F0 mosaic cuts were confirmed with gel electrophoresis and sequencing, with clutches having confirmed CRISPR cuts grown to adulthood. Adult F0 CRISPR-injected fish were screened for germline mutation by outcrossing and testing individual larvae using the primers above and sequencing. Heterozygous *rhbdl2*^*+/−*^ fish were maintained by outcrossing F1 CRISPR mutants to AB WT background zebrafish before being genotyped by genomic DNA extraction from fin clips. PCR was carried using the primers listed above along with an additional set to detect deletion of the genomic region of interest: R2 Mutant F: 5’ – GAATTAAGTTACCCGTTTATGTG – 3’, and R2 Mutant R: 5’ – CTTTGCAGAAGGACGAAGAAG – 3’. PCR products were separated on a 1.5% agarose gel for 30 min to determine individual fish genotypes.

For experimental purposes, F2 or F3 heterozygotes were incrossed to produce a generation of adult homozygous WT and mutant siblings. These adults were incrossed to produce WT and *rhbdl2*^*−/−*^ clutches. These cousin larvae were only used for experimentation, while outcrossing of heterozygotes continued to be used to maintain subsequent generations.

### RT-qPCR

RNA and DNA were extracted from pooled samples of 10–20 larvae from homozygous incrosses using TRIZOL (Invitrogen) and following the manufacturer’s protocol. DNA underwent PCR amplification using Q5 High-Fidelity 2X Master Mix (New England Biolabs) as described above to confirm genotypes. Extracted RNA was used for cDNA production using High-Capacity cDNA Reverse Transcription Kit (Applied Biosciences). qPCR was performed using AzuraView GreenFast qPCR Blue Mix LR (Azura Genomics) and an OpusFlex384 (Bio-Rad). Primers for target genes are listed below. Data were normalized to *rps11* using the ΔΔCt method (Livak 2001) to illustrate fold change over WT samples.

For wound induced expression analysis of *rhbdl2,* incrosses of homozygous adult WT and *rhbdl2*^*−/−*^ siblings were done to produce homozygous cousins of WT and mutants. Larvae at 2dpf were injured with a transection wound at the tip of the notochord. At 24hpw, fins were collected by transection of regenerating tissue at the tip of the blood circulatory loop. 30–50 fins were pooled and flash frozen, with unwounded 3dpf control tissue collected at the same stage. RNA was extracted from fin tissue as described above. Primers for *rhbdl2* and *rps11* are listed below and data were normalized to *rps11* and represented as fold chang over unwounded WT, age-matched control tissue.

RT-qPCR primers were used as followed: Rhbdl2 exon4 qF: 5’ – CATAAAGGCTTTGAAGTTGG – 3’, Rhbdl2 exon4 qR: 5’ – CATAAGGGCATAAACACCAC – 3’ ; Rhbdl1 exon2 qF: 5’ – GTACAGATCATGGTGTTCAT – 3’, Rhbdl1 exon2 qR: 5’ – GCAGCACCCATTTATTGAGC – 3’ ; Rhbdl3 exon1 qF: 5’ – GTAGCTCCAGAGGACCAATG – 3’, Rhbdl3 exon1 qR: 5’ - CAGTTCTGATCCATGAGCTG – 3’ ; Rhbdl4 exon4 qF: 5’ – ACCCTGGGACATCATTTGTT – 3’, Rhbdl4 exon4 qR: 5’ – CCCTCCATAATGCCCATTAG – 3’ ; rac1a qF: 5’ – TCCGGCCTCATTTGAAAACG – 3’, rac1a qR: 5’ – GTGTCCTTGTCATCTCGCAA – 3’ ; rac1b qF: 5’ – CTCTCTCGTACCCTCAGACG – 3’, rac1b qR: 5’ – TGGGAGTGGTTTGACAGTGA – 3’ ; rac2 qF: 5’ – AATACCTGGAGTGTTCGGCC – 3’, rac2 qR: 5’ – GCCCTTCTTCTTGACCTTGG – 3’ ; rac3a qF: 5’ – CCCAGGAGAGTATATACCCACAG – 3’, rac3a qR: 5’ – GGTATGAAAGTGGTCTAAGGCG – 3’ ; rac3b qF: 5’ – CCCCGGCGAGTACATCCCTACAG – 3’, rac3b qR: 5’ – GGTATGAGAGTGGACGGAGGCG – 3’ ; rps11 qF: 5’ - TAA GAA ATG CCC CTT CAC TG – 3’, rps11 qR: 5’ - GTC TCT TCT CAA AAC GGT TG – 3’.

### Developmental Imaging

Homozygous cousin larvae were generated from adult WT and *rhbdl2*^*−/−*^ sibling as described above. Larvae were raised to 2dpf without PTU and anesthetized before imaging on a Zeiss Discovery.V8 stereoscope with an Apo S 1.0X FWD 60 mm objective (Zeiss). Images were taken at 3.2X magnification using an Axiocam 208 color camera (Zeiss) and Labscope version 4.2.1 (Zeiss). Adult fish were raised to 6mpf and separated by sex. Adult fish were anesthetized in Tricaine (0.017% in E3 media) and placed on petri dish with white tape underneath for best visualization. Water was removed to leave a small amount to cover the fish and allow for respiration. Fish were briefly imaged using an iPhone 13 camera with a ruler placed adjacent for size measurements.

### Regeneration assays

For larval regeneration assays, incrosses of adult F3 WT and *rhbdl2*^*−/−*^ siblings were set up to produce homozygous cousins possessing WT or promoterless alleles. Larvae were dechorionated at 2dpf and transferred to petri dishes previously coated in milk. Tail transections were performed at 3dpf using a surgical blade (feather no.10) to create a vertical cut at the posterior tip of the developing notochord in the caudal fin without injuring the notochord. Larvae were washed in E3 and allowed to regenerate for up to 4 days post-wounding (dpw). At indicated times, larvae were fixed with 4% PFA (Sigma-Aldrich) in PBS overnight at 4°C, followed by washing three times in PBS. Larvae were prepared for imaging by bisecting larvae at the posterior tip of the yolk sac in order for fins to lay flat before transferring to a 35 mm glass bottom imaging dish (Willco). Fins were imaged in PBS at RT with an AX-R point-scanning confocal on a Ti2-E inverted microscope (Nikon). Images were acquired using a 20X Plan Apo Lambda D objective (Nikon) as 50μm z-stacks with 5-μm optical sections and 512 × 512 resolution. Images were acquired and processed using NIS Elements Ar version 6.10.01. Unwounded age-matched larvae at 6dpf were measured as a developmental control.

### EdU labeling and immunohistochemistry

Adult F3 WT and *rhbdl2*^*−/−*^ siblings were incrossed to produce homozygous cousin offspring. Proliferation in the fin was measured with the Click-iT Plus EdU Imaging Kit (Life Technologies). After transection wounding as described above, wounded larvae were incubated in a 250 μM EdU solution in E3 media for 6h at RT. Wounded larvae were incubated from 66–72hpw alongside age-matched unwounded controls. Larvae were fixed overnight in 4% PFA in PBS at 4°C before storage in methanol at −20°C until staining. Larvae were rehydrated in a series of 5-min washes at ratios of 2:1, 1:1, and 1:2 methanol: PBSTx (0.2% Triton X-100 in PBS) followed by 30 min washes twice in PBSTx. Larvae were blocked in a 1.5% sheep serum, 1% BSA, 0.2% Triton X-100 solution in PBS for 3 hr before being stained according to manufacturer’s instructions overnight at 4°C in the dark. All incubations included slight agitation by rocking. Transection of tails at yolk sac and imaging in glass bottom dish was done as described above. Immunofluorescence images were acquired using an AX-R point-scanning confocal on a Ti2-E inverted microscope (Nikon) equipped with an LUA-S4 laser unit with 405/488/561/640 lines. Images acquired with 20X air objective (50 μm z-stacks, 3-μm optical sections, and 512 × 512 resolution).

### Mitosis and apoptosis labeling

Homozygous cousin larvae were generated and wounded at 3dpf as previously described. Larvae were fixed at either 24hpw or 72hpw in 4% PFA in PBS overnight at 4°C before transfer to methanol at −20°C. Larvae were rehydrated in a series of 5-min washes at ratios of 2:1, 1:1, and 1:2 methanol: PDT solution (0.3% Triton-X, 1% DMSO, 0.05% Tween-20 in PBS) followed by washing twice in PDT for 30 min at RT. Larvae were blocked in a solution of 10% heat-inactivated FBS, 2% BSA, 0.05% Tween-20 in PBS for 1 hr at RT. Samples were costained with monoclonal rabbit anti-active Caspase3 antibody (#559565, BD Biosciences) diluted 1:500 and monoclonal rat anti-phospho-H3 (pSer 28) antibody (H9908, Sigma-Aldrich) diluted 1:300 in blocking overnight at 4°C. Samples were washed twice in PDT for 30 min at RT and incubated in blocking again for 1 hr. Incubation with Dylight donkey anti-rabbit 550 and Dylight donkey anti-rat 488 secondary antibodies diluted 1:300 was performed overnight at 4°C. Samples were washed three times in PDT before imaging in a glass-bottom dish using the laser scanning microscope with Plan-apochromat NA 0.8/20X air objective, as described above.

### Live Rhobo6 Staining and Imaging

Homozygous cousin larvae were generated and wounded at 3dpf as previously described. Wounded larvae were grown to 72 hpw whereby they were anesthetized and loaded onto a plate of 3% agarose in E3/MB with a small amount of water. Rhobo6 dye was prepared and diluted to 1mM in DMSO. A final solution at 100μM was made with a 1:9 mixture of dye with phenol red and 3 nL were injected into the caudal vein of the 72hpw larvae. Confirmation of injection was detected by the presence of phenol red in the heart of larvae. Larvae were allowed to recovery for 30 min in E3/MB supplemented with Rhobo6 (5μM). Larvae were mounted on their side in 3% low-melting point agarose with tails open by manually removing agarose around area. Dishes were covered in E3 and mounted 10–20 min before performing two-photon imaging on a ThorLabs Bergamo II multiphoton microsocope and a fs-pulsed 80-Mhz Ti:S laser (MaiTai DeepSee; SpectraPhysics). Live images were captured using an excitation wavelength of 1080 nm (<10 mW of excitation power) and a 16X/0.8W Nikon water immersion objective (CF175 LWD). Imaging was controlled by ThorImage 4.3. Frames were 1024 × 512 pixels covering a 454.75 × 232.38 μm field of view (1.8X magnification) over 50–80 μm in Z, collected at a rate of 30Hz, and averaged forty times to increase signal to noise. Images were subsequently processed in ImageJ to produce average z-stacks over the entire Z-stack and then blinded and scored in ImageJ.

### Mass Spectroscopy

#### Sample preparations:

Samples were desiccated and reconstituted in 200ul of 6M Guanidine -HCl. The samples were then boiled for 10 minutes followed by 5 minutes cooling at room temperature. The boiling and cooling cycle was repeated a total of 3 cycles. The proteins were precipitated with addition of methanol to final volume of 90% followed by vortex and centrifugation at maximum speed on a benchtop microfuge (14000 rpm) for 10 minutes. The soluble fraction was removed by flipping the tube onto an absorbent surface and tapping to remove any liquid. The pellet was suspended in 200ul of 8 M Urea made in 100mM Tris pH 8.0. TCEP was added to final concentration of 10 mM and Chloro-acetamide solution was added to final concentration of 40 mM and vortex for 5 minutes. 3 volumes of 50mM Tris pH 8.0 were added to the sample to reduce the final urea concentration to 2 M. Trypsin was in 1:50 ratio of trypsin and incubated at 37C for 12 hours. The solution was then acidified using TFA (0.5% TFA final concentration) and mixed. The sample was desalted using C18-StageTips (Thermo) as described by the manufacturer protocol. The peptide concentration of sample was measured using BCA.

#### Mass spec acquisition:

DDA; 1 ug of each sample was analyzed by ultra high pressure liquid chromatography (UPLC) coupled with tandem mass spectroscopy (LC-MS/MS) using nano-spray ionization. The nanospray ionization experiments were performed using a TimsTOF 2 HT hybrid mass spectrometer (Bruker) interfaced with nano-scale reversed-phase UPLC (EVOSEP ONE). Evosep method 15 samples per day was utilized using a 15 cm × 150 μm reverse-phase column packed with 1.5 μm C18-beads (PepSep, Bruker) at 58 °C. The analytical columns were connected with a fused silica ID emitter (10 μm ID; Bruker Daltonics) inside a nanoelectrospray ion source (Captive spray source; Bruker). The mobile phases comprised 0.1% FA as solution A and 0.1% FA/99.9% ACN as solution B. The mass spectrometry setting for the TimsTOF Pro 2 are as following: PASEF method for standard proteomics. The values for mobility-dependent collision energy ramping were set to 95 eV at an inversed reduced mobility (1/k0) of 1.6 V s/cm2 and 23 eV at 0.73 V s/cm2. Collision energies were linearly interpolated between these two 1/k0 values and kept constant above or below. No merging of TIMS scans was performed. Target intensity per individual PASEF precursor was set to 20 000. The scan range was set between 0.6 and 1.6 V s/cm2 with a ramp time of 166 ms. 14 PASEF MS/MS scans were triggered per cycle (2.57 s) with a maximum of seven precursors per mobilogram. Precursor ions in an m/z range between 100 and 1700 with charge states ≥3+ and ≤8+ were selected for fragmentation. Active exclusion was enabled for 0.4 min (mass width 0.015 Th, 1/k0 width 0.015 V s/cm2). Peptide identification were carried out using Peaks Studio 12.5 (Bioinformatics solutions Inc.)

#### Mass spectrometry analysis:

To evaluate proteomic changes, adult F3 WT and *rhbdl2*^*−/−*^ siblings were incrossed to produce homozygous cousin offspring. Larvae were raised without PTU until 2dpf before anesthetization. 50–60 larvae at 2dpf were pooled and deyolked in Ringer’s solution with gentle disruption using a p1000 pipette tip. Larvae were washed twice in PBS and solution was removed before samples were stored at −80°C until submission for LC-MS/MS analysis. DB values for each protein were generated for three replicates each of both wild-type and *rhbdl2*^*−/−*^ samples. These estimated abundance measures were merged across all samples and used as input to the Perseus software package (Tyanova et al., 2016) for processing. Abundance values were log-transformed and missing values for proteins in samples where the protein was undetected were imputed. Imputed values were assigned using the Perseus default method which randomly assigns values from a Gaussian distribution with a −0.3 sd downshift. Lastly, the values were normalized using a “width adjustment” before being exported to the R statistical environment (www.cran.org). Within R, the data were used in differential abundance analysis using the limma package (Ritchie et al., 2015) to compare the WT and *rhbdl2*^*−/−*^ groups. Significantly differentially abundant proteins (p-value < 0.05) were used as input for enrichment analysis in the Gene Ontology, KEGG, and Reactome ontologies with the gProfiler tool (https://biit.cs.ut.ee/gprofiler/gost, Kolberg, et al., 2020). Scatter plots and box plots were made with the ggplot2 package in R while the heatmap were made with the gplots package.

### Leukocyte imaging

For analysis of leukocytes, a transgenic line was developed by crossing *rhbdl2*^*+/−*^ to AB WT zebrafish labeled with fluorescent neutrophils (*Tg(lyz:GFP))* or fluorescent neutrophils and macrophages (*Tg(lyz:GFP;mpeg1:dsRed))*. Genotyped heterozygotes were incrossed to produce homozygous, fluorescently labeled adults that were incrossed again to produced WT and *rhbdl2*^*−/−*^ cousins for experimental use. Wounding and fixing was performed as described above before fins were imaged on the laser scanning confocal as described for regeneration assays. Whole larvae were live imaged in a zWEDGI (Huemer 2017) at indicated stages and acquired on tthe point-scanning confocal with a 10X plan Apo Lambda D air objective (180 μm z-stacks, 5μm optical sections, 3 × 1 tiles with 15% overlap, 512 × 512 resolution). For time-lapse imaging of leukocyte recruitments, 3dpf WT and *rhbdl2*^*−/−*^ larvae were wounded by tail transection as described above. Larvae were mounted in zWEDGI devices (held in place using 1% low-melting-point agarose (Sigma-Aldrich) at the head). Images of caudal fin were acquired at RT using the point-scanning confocal microscope (20X Plan Apo Lambda D air objective, 50 μm z-stacks, 5μm optical sections, one z-stack every 2 min for 6h at indicated times following injury, 512 × 340 resolution).

### Morpholino Injections

Previously published morpholinos were obtained from GeneTools for *rac2*, 5’ - CCACCACACACTTT ATTGCTTGCAT – 3’ (Deng 2012) along with the standard control MO (Genetools). Morpholinos were resuspended in water to a stock concentration of 1mM. Morpholinos were further diluted in water with 0.5X CutSmart Buffer (New England Biosciences) and 0.1% phenol red to an injected concentration of 0.1 mM. Alongside the same concentration of control MO, 1 nL of diluted morpholino was injected at the 1-cell stage of fertilized embryos.

### Image analysis and processing

All images were acquired at RT and processed using NIS Elements Ar version 6.10.01. Images are always shown with anterior to the left and dorsal at the top except where noted. Brightness and contrast were adjusted for best visualization and were not used during fluorescent analysis.

#### Regeneration area quantification

Regenerated area was measured from the posterior tip of the notochord to the caudal edge of the tail fin with the polygon tool in FIJI image analysis software (Schindelin et al. 2012).

#### Immunohistochemistry

For EdU analysis, images were reconstructed in 3D using Imaris software (Bitplane). The Spots function was used to quantify the number of EdU-positive cells in the fin region distal to the blood circulatory loop, where xy = 7μm and z = 14μm. Phospho-H3 positive cells were counted manually using max projected images in the same area beyond the blood loop. Apoptosis activation was quantified on max projected images in FIJI by outlining the fin past the blood circulatory loop using the polygon tool in the corresponding brightfield image. Total threshold area for active-Casp3 signal was determined using the threshold plugin in FIJI by auto-thresholding with the “Yen Dark” method (Yen et al 1995).

#### Leukocyte Counts

Leukocyte numbers in wound assays were determined by hand in the region beyond the caudal vein tip using FIJI software on maximum intensity projections. Total neutrophil and macrophage numbers were determined using Imaris (Bitplane) with the Spots function defined by xy = 10μm and z = 20 μm. Leukocytes within the eye and heart were excluded. Full size measurements were also taken on max projected images for 2dpf length. Using time-lapse confocal images, leukocytes were tracked using the Spots function. Threshold for spot size was set at xy=10 for neutrophils and xy=8 for macrophages. The cells recruited to the wound area distal to the caudal vein loop were tracked manually.

### Statistical analyses

All graphs were plotted on Graphpad (Prism v. 10). For all statistical analyses, at least three independent replicates were conducted, with a replicate defined as a separate clutch of larvae spawned on different days. RT-qPCR results were determined by comparing the calculated ΔCq values of the experimental conditions using a one-sample t-test. Fold change was calculated and plotted in terms of mean with 95% confidence interval (CI). Differential protein abundances were compared using the limma package within R (Ritchie et al., 2015). For all other quantitative experiments, data were pooled from independent replicates and summarized in terms of least-squared adjusted means (emeans) and standard error of the mean (Vincent et al., 2016, Golenberg et al., 2020). These results were analyzed using Anova with a Tukey’s multiple comparisons test. Graphical representation depicts individual data points color coded to reflect replicates. Averages per replicate represented by solid color points superimposed on graphs. Statistical analysis and graphical representations were done using R version 4.5.2 and GraphPad prism version 10.

## Supplementary Material

Supplement 1

## Figures and Tables

**Table 1.** Annotated RHBDL2 sequences used in this study.

Name	Sequence ID	Database
zRhbdl2 205	ENSDART00000183149.1	ENSEMBL
zRhbdl2 203	ENSDART00000138244.3	ENSEMBL
zRhbdl2 202	ENSDART00000101953.4	ENSEMBL
zRhbdl2 204	ENSDART00000179385.2	ENSEMBL
zRhbdl2 201	ENSDART00000058471.7	ENSEMBL
hRhbdl2 (RHBL2_HUMAN)	Q9NX52	UniprotKB
zRhbdl2 (F1QAX9_DANRE)	F1QAX9	UniprotKB

## Data Availability

The proteomics datasets generated during this study are available in the Mendeley Data repository at DOI: 10.17632/gwh3j3rrjr.1.
