# Supplementary material for: Rhomboid protease Rhbdl2 regulates macrophage recruitment and wound regeneration in zebrafish
Source: bioRxiv. 2026 Feb 15:2026.02.13.705804. Preprint. [Version 1] doi: 10.64898/2026.02.13.705804 (PMC12918973; doi:10.64898/2026.02.13.705804)
Supplement: Supplement 1 [file NIHPP2026.02.13.705804v1-supplement-1.pdf]

bioRxiv preprint doi: https://doi.org/10.64898/2026.02.13.705804; this version posted February 15, 2026. The copyright holder for this preprint (which was not certified by peer review) is the author/funder, who has granted bioRxiv a license to display the preprint in perpetuity. It is made available under aCC-BY 4.0 International license.

Supplemental Figure Legends

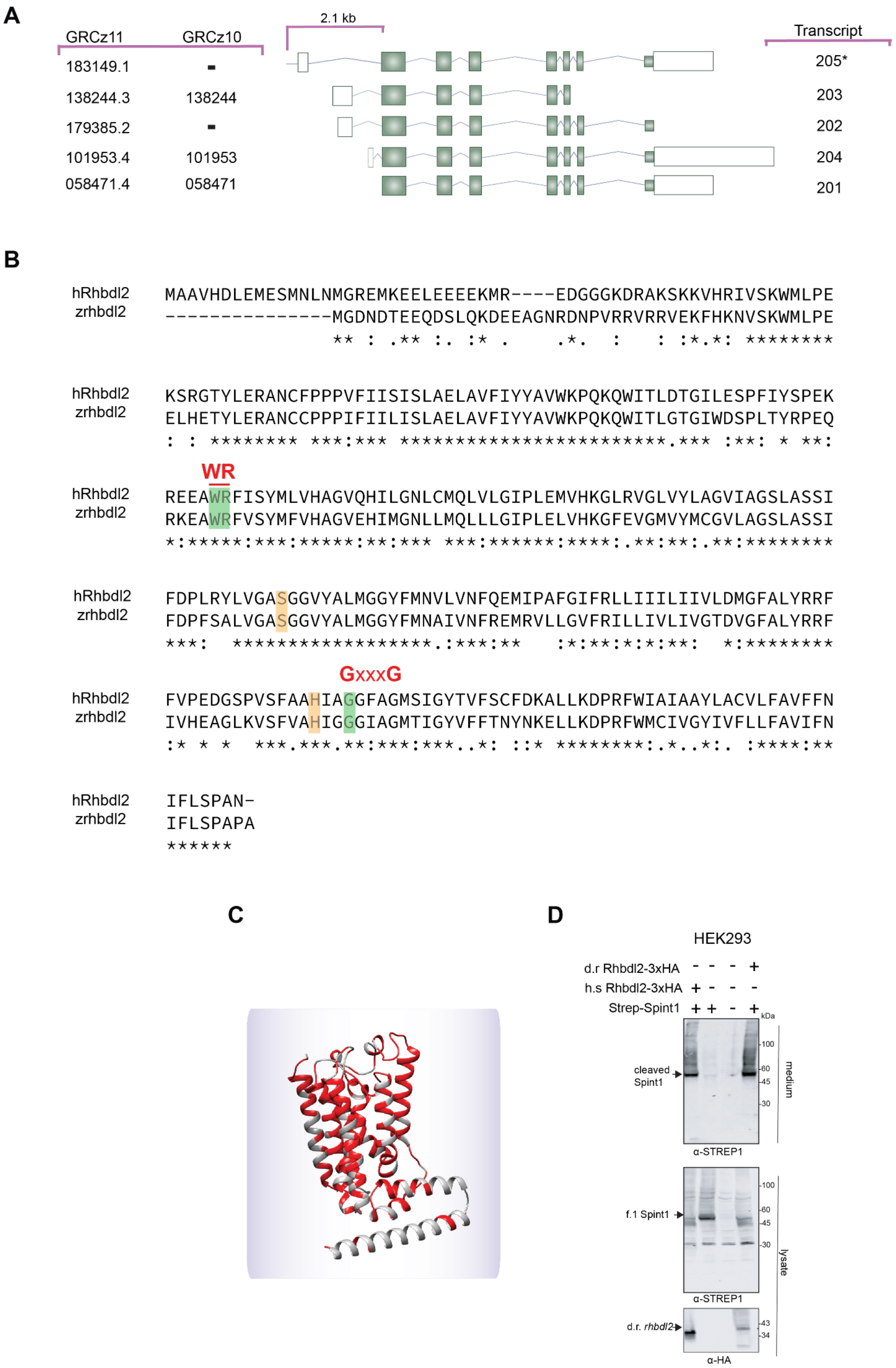

Fig. S1. Characterization of zebrafish Rhbdl2 structure and function. (A) Diagrams of *rhbdl2* transcripts, where exons are displayed as solid boxes while introns are the connecting lines. Left: A list of ENSEMBL IDs with the last seven digits from both GRCz11 and GRCz10 genome assemblies (full accession IDs listed in Materials and Methods). Right: List of transcript reference

bioRxiv preprint doi: https://doi.org/10.64898/2026.02.13.705804; this version posted February 15, 2026. The copyright holder for this preprint (which was not certified by peer review) is the author/funder, who has granted bioRxiv a license to display the preprint in perpetuity. It is made available under aCC-BY 4.0 International license.

names from GRCz11. Asterisk indicates canonical transcript discussed in this paper while arrows highlight the sites of CRISPR-Cas9 guide mutagenesis, with the distance between indicated. **(B)** Amino acid sequence comparison between human Rhbdl2 and zebrafish Rhbdl2 (transcript 205). Conserved rhomboid motifs (WR and GxxxG) are highlighted in green and the serine-histidine catalytic dyad residues are highlighted in orange. **(C)** Alphafold model depicting overlap between human and zebrafish Rhbdl2. Conserved residues are displayed in red. **(D)** Western blot of transiently transfected HEK293T cell culture conditioned media and lysate probed with Strep- (Spint1) and HA- (Rhbdl2) antibodies. The top blot detects Spint1 ectodomain shed by cells while middle and bottom blots display Spint1 and Rhbdl2 detection in lysates. N=3 replicates.

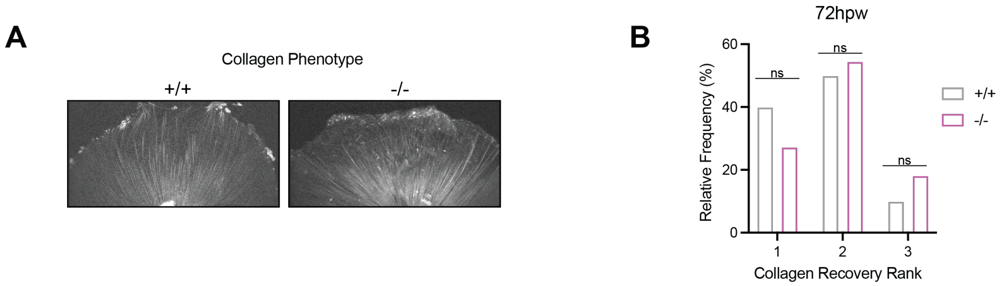

Fig. S2. Characterization of the ECM matrix in regenerated fins of *rhbdl2$^{-/-}$* mutants. (A) Representative images of WT or *rhbdl2$^{-/-}$* regenerated fins at 72hpw and labeled with Rhobo6. Images are average fluorescent z-stacks from two-photon microscopy, and levels were adjusted for best visualization. (B) Collagen regeneration scoring using a blinded phenotypic screening system across both genotypes as follows: 1 = little to no aberrations, 2 = moderate irregularities and scarring in collagen matrix, 3 = large irregularities or missing areas of signal. Data is from three independent experiments, n = 10 (+/+), 11 (-/-). Two-way ANOVA was conducted (p = 0.6667) and scale bar = 100 µm.

bioRxiv preprint doi: https://doi.org/10.64898/2026.02.13.705804; this version posted February 15, 2026. The copyright holder for this preprint (which was not certified by peer review) is the author/funder, who has granted bioRxiv a license to display the preprint in perpetuity. It is made available under aCC-BY 4.0 International license.

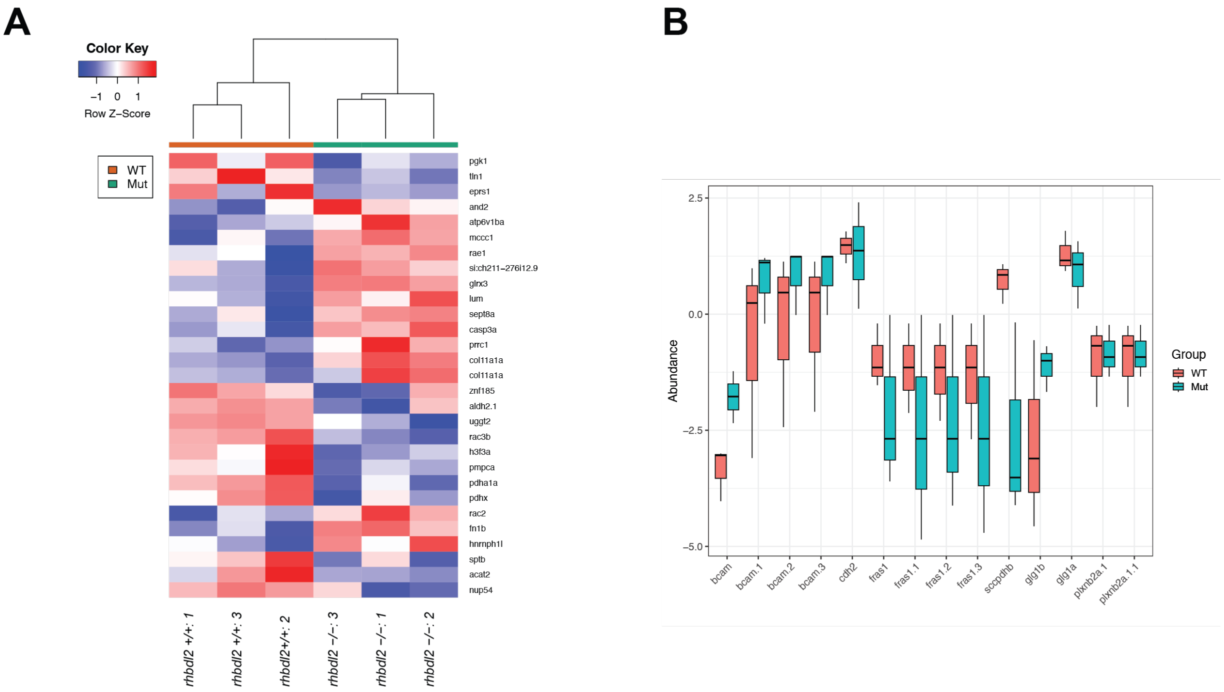

Fig. S3. Differential abundance of proteins between WT and *rhbdl2*$^{-/-}$ larvae. (A) Heatmap of differentially abundant proteins that are detected in all WT and *rhbdl2*$^{-/-}$ samples. WT (orange) and *rhbdl2*$^{-/-}$ (teal) samples are samples are represented across the top along with a dendrogram showing the clustering of the samples based on the abundance of the proteins shown. **(B)** Boxplot of the proteins overlapping between the mass spectrometry proteomics results and Table 1 of Johnson, et al. For each protein, the proteomic abundance is shown for the WT (orange) and *rhbdl2*$^{-/-}$ samples (teal). The box represents the interquartile range (IQR, 25%-75%) with the median represented by a black line. The whiskers represent the range +/- 1.5x IQR.

bioRxiv preprint doi: https://doi.org/10.64898/2026.02.13.705804; this version posted February 15, 2026. The copyright holder for this preprint (which was not certified by peer review) is the author/funder, who has granted bioRxiv a license to display the preprint in perpetuity. It is made available under aCC-BY 4.0 International license.

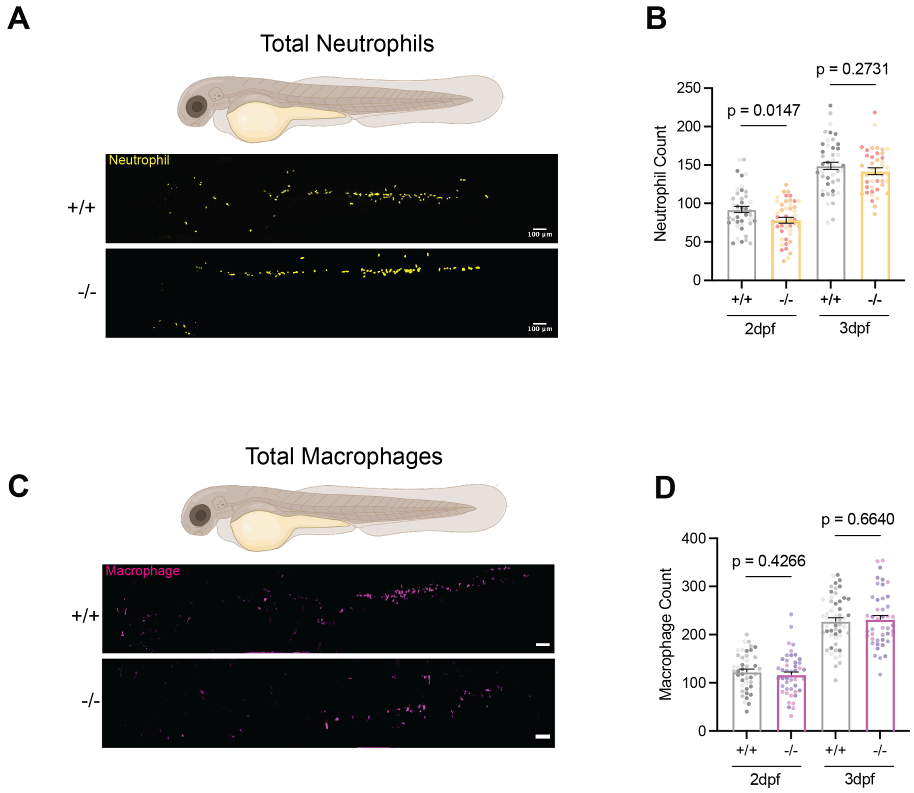

Fig. S4. Development of leukocytes is unaffected in promoterless *rhbdl2*$^{-/-}$ mutants. (A) Representative full body images of 2dpf whole larvae with GFP-labeled neutrophils. **(B)** Quantification of total number of neutrophils in whole larvae at 2dpf and 3dpf from three independent replicates (2dpf, n = 43 (+/+), 46 (-/-) ; 3dpf, n = 45 (+/+), 45 (-/-)). **(C)** Representative full body images of 2dpf whole larvae with dsRed-labeled macrophages. **(D)** Quantification of total number of macrophages in whole larvae at 2dpf and 3dpf from three independent replicates (2dpf, n = 44 (+/+), 44 (-/-) ; 3dpf, n = 52 (+/+), 44 (-/-)). Lsmeans (± SEM) reported and p-values calculated by ANOVA with Tukey's multiple comparisons for all experiments. Scale bar = 100 µm.
